# AI-based analysis of fetal growth restriction in a prospective obstetric cohort quantifies compound risks for perinatal morbidity and mortality and identifies previously unrecognized high risk clinical scenarios

**DOI:** 10.21203/rs.3.rs-5126218/v1

**Published:** 2024-12-16

**Authors:** Raquel M. Zimmerman, Edgar J. Hernandez, Mark Yandell, Martin Tristani-Firouzi, Robert M. Silver, William Grobman, David Haas, George Saade, Jonathan Steller, Nathan R. Blue

**Affiliations:** University of Utah Health; University of Utah Health; University of Utah Health; University of Utah Health; University of Utah Health; Brown University; Indiana University; Eastern Virginia Medical School; University of California, Irvine; University of Utah Health

**Keywords:** Explainable artificial intelligence, pregnancy, fetal growth restriction, stillbirth, perinatal morbidity

## Abstract

**Background:**

Fetal growth restriction (FGR) is a leading risk factor for stillbirth, yet the diagnosis of FGR confers considerable prognostic uncertainty, as most infants with FGR do not experience any morbidity. Our objective was to use data from a large, deeply phenotyped observational obstetric cohort to develop a probabilistic graphical model (PGM), a type of “explainable artificial intelligence (AI)”, as a potential framework to better understand how interrelated variables contribute to perinatal morbidity risk in FGR.

**Methods:**

Using data from 9,558 pregnancies delivered at ≥ 20 weeks with available outcome data, we derived and validated a PGM using randomly selected sub-cohorts of 80% (n = 7645) and 20% (n = 1,912), respectively, to discriminate cases of FGR resulting in composite perinatal morbidity from those that did not. We also sought to identify context-specific risk relationships among inter-related variables in FGR. Performance was assessed as area under the receiver-operating characteristics curve (AUC).

**Results:**

Feature selection identified the 16 most informative variables, which yielded a PGM with good overall performance in the validation cohort (AUC 0.83, 95% CI 0.79–0.87), including among “N of 1” unique scenarios (AUC 0.81, 0.72–0.90). Using the PGM, we identified FGR scenarios with a risk of perinatal morbidity no different from that of the cohort background (e.g. female fetus, estimated fetal weight (EFW) 3–9th percentile, no preexisting diabetes, no progesterone use; RR 0.9, 95% CI 0.7–1.1) alongside others that conferred a nearly 10-fold higher risk (female fetus, EFW 3–9th percentile, maternal preexisting diabetes, progesterone use; RR 9.8, 7.5–11.6). This led to the recognition of a PGM-identified latent interaction of fetal sex with preexisting diabetes, wherein the typical protective effect of female fetal sex was reversed in the presence of maternal diabetes.

**Conclusions:**

PGMs are able to capture and quantify context-specific risk relationships in FGR and identify latent variable interactions that are associated with large differences in risk. FGR scenarios that are separated by nearly 10-fold perinatal morbidity risk would be managed similarly under current FGR clinical guidelines, highlighting the need for more precise approaches to risk estimation in FGR.

## Background

Fetal growth restriction (FGR) is commonly defined as estimated fetal weight (EFW) < 10th percentile and is a leading risk factor for stillbirth.^1^ While FGR is an important indicator of stillbirth risk, most fetuses diagnosed with FGR do not experience any morbidity, such that the diagnosis of FGR confers a wide range of possible perinatal outcomes, from wellness to severe illness or death.^2–4^ For both patients and clinicians, the high degree of uncertainty inherent in a prenatal diagnosis of FGR poses difficulties for care planning, underscoring the crucial importance of effective risk stratification for quality perinatal care. Although multivariable modeling has been studied as a potential means to improve prediction of adverse outcomes in FGR, significant progress has not been made in accurately identifying risk strata.^5^

Artificial intelligence (AI)-based technologies hold promise to improve estimation of perinatal morbidity risk in FGR. However, concerns exist about the transparency of AI analyses.^6–8^ Reverting to human risk estimation does not resolve these concerns, as human cognition is also opaque, state-dependent, and prone to bias.^9–11^

“Explainable AI” describes a subset of AI methodologies designed to improve trustworthiness by providing interpretable, transparent explanations for model decisions and predictions, thus allowing for human intellectual oversight and critical evaluation of outputs.^12^ Probabilistic graphical models (PGMs) are a type of explainable AI that are well-suited to risk stratification because of their ability to capture and quantify conditional dependencies between interrelated variables in a transparent manner.^13,14^ Our objectives were 1) to derive a PGM to refine risk estimation for composite perinatal morbidity and mortality and 2) to identify clinical contexts, or combinations of variables that are associated with increased or reduced morbidity risk in FGR.

## Methods

This study uses data from the NICHD-funded Nulliparous Pregnancy Outcomes Study: monitoring mothers-to-be (nuMoM2b) observational cohort.^15^ The nuMoM2b dataset consists of 10,038 nulliparous participants with ultrasound-confirmed, viable gestations who were recruited between 6 weeks + 0 days and 13 weeks + 6 days’ gestation. Recruitment took place at eight geographically diverse U.S. centers between October 2010 and September 2013. Participants were eligible for inclusion if they had no prior pregnancies reaching at least 20 weeks’ gestation. Exclusion criteria included age < 13 years, history of ≥ 3 pregnancy losses, the presence of a likely fatal fetal malformation already evident at enrollment screening, known fetal aneuploidy, conception using a donor oocyte, multifetal reduction, plan to terminate pregnancy, or participation in an intervention study. Participants were followed longitudinally, undergoing four study visits at the following gestational ages: Visit 1, 6w0d to 13w6d; Visit 2, 16w0d to 21w6d; Visit 3, 22w0d to 29w6d: Visit 4, after delivery. Study visits involved detailed interviews, questionnaires, research ultrasounds, maternal biometric measurements, and biospecimen collection. Ultrasounds were performed at all three study visits during pregnancy and included experimental as well as standard clinical assessments. Details of medication use, medical history, and family medical history were ascertained during interviews.

In addition to clinical parameters, data collection included validated instruments that assessed multiple psychosocial domains, such as stress, social support, shift-work timing, pregnancy intendedness, and experiences of discrimination. After delivery, pre-specified obstetric, maternal, and neonatal outcomes were ascertained through maternal interviews and medical record abstraction by centrally trained research personnel. Methods of the nuMoM2b study and data collection have been described in detail.^16^

All nuMoM2b participants who delivered ≥ 20 weeks’ gestation with available birth and neonatal outcomes data were included in this analysis. The only exclusion criteria were delivery prior to 20 weeks and missing birth and neonatal outcome data. The primary clinical outcome for this analysis was a composite of perinatal morbidity and mortality that was selected to reflect the consequences of fetal or neonatal compromise that sonographically identified FGR is supposed to identify. Composite morbidity was defined by the presence of any of the following factors: stillbirth, neonatal death, the need for mechanical ventilation, respiratory distress syndrome (RDS), necrotizing enterocolitis (NEC), confirmed sepsis, grade 3 or 4 intraventricular hemorrhage (IVH), neonatal seizures, or NICU admission greater than seven days. These factors were selected from the FGR core outcome set outlined in the COSGROVE study.^17^ We used a random subset of 80% of the 10,038 nuMoM2b participants for model derivation. The remaining 20% were used for model validation.

### Feature selection

The initial set of potentially eligible features included > 4000 unique variables. After expert review, these were pruned to a set of 907 candidate variables, which included variables from multiple domains: clinical (e.g., blood pressure, BMI, fetal sex, maternal hospitalizations during pregnancy, medication use, mode of delivery); ultrasound (visit 3 estimated fetal weight (EFW) percentile, visit 2 uterine artery pulsatility index, visit 2 cervical length); psychosocial (poverty, education level, experiences of discrimination, Perceived Stress scale, Edinburgh Postnatal Depression scale, State-Trait Anxiety Index-Trait scale, Multidimensional Scale of Perceived Social Support); and multi-substance quantitative urine toxicology performed for research purposes.^18–22^ Missingness was not used to exclude variables, as PGMs inherently handle missing data by modeling the “unknown” state for each analyzed variable.

When possible, continuous variables were dichotomized using established clinically relevant definitions (e.g. blood pressure, cervical length). Continuous variables for which values may be associated with adverse outcomes at both upper and lower extremes (e.g. maternal age, BMI) were collapsed into multiple categories using established definitions, which were then one-hot encoded such that each category was tested as a separate dichotomous variable (present/absent). Some variables, such as hypertensive disorders of pregnancy, were tested both as dichotomized and as one-hot encoded sub-categories (gestational hypertension, mild preeclampsia, severe preeclampsia, HELLP syndrome, eclampsia, unspecified) and were selected for further testing based on the strength of their association. Continuous variables without established thresholds were assessed for association with the outcome using logistic regression, and empirical thresholds were established using receiver operating characteristics (ROC) methodology. Ultimately, fetal growth was categorized as EFW < 3rd percentile, EFW 3–9th percentile, EFW 10–90th percentile, or EFW > 90th percentile by study ultrasound done at visit 3 (22–29w6d) and using Hadlock EFW percentiles. Gestational age at birth was categorized by standard definitions of early preterm birth (PTB, < 34 weeks) late PTB (34–36 weeks), and term birth (≥ 37 weeks). Hypertensive disorders of pregnancy (HDP) were empirically dichotomized based on association with perinatal morbidity, such that the dichotomized “HDP” variable was ultimately defined as either having eclampsia, preeclampsia with severe features, or gestational hypertension with onset prior to labor.

We chose to include all possible variables that would explain variation in perinatal morbidity risk, including those that could not be known until the time or birth, because there currently is no gold standard for FGR against which to benchmark prediction models. The adverse outcomes caused by FGR (stillbirth, perinatal morbidity, preterm birth, etc.) often occur via multiple other pathologies, and the lack of a diagnostic gold standard for FGR means it is unclear what proportion of such adverse perinatal outcomes are directly attributable to FGR. As a result, even an excellent FGR-focused model is unlikely to achieve good overall performance if it does not account for alternate causes of perinatal morbidity. This is likely the reason why studies comparing various diagnostic strategies for FGR, including FGR-specific multivariable models, have yielded statistically significant but clinically insignificant improvements in overall morbidity risk stratification performance,^23–29^ a finding our group has replicated repeatedly.^2,4,5,30,31^. Therefore, we included all variables that might account for perinatal morbidity risk in order to better capture and understand the landscape of factors that drive risk in FGR.

Candidate features for a logistic regression (LR) model were identified with chi-square analysis and mutual information criterion analysis. MIC quantifies the information shared between possible predictors and the target variable, and chi-square assesses independence between the possible predictor and target variable. We used a Jaccard similarity matrix to reduce redundancy favoring variables with standard clinical definitions, when possible, to maximize clinical interpretability. The 30 variables demonstrating the strongest association with the composite primary outcome by chi-square and MIC were selected. Because computational constraints limit exact PGMs to less than 20 variables, we then created all possible combinations of 12 variables from the top 30 and derived a logistic regression model for each. We chose the set of 12 variables with the best-performing LR model based on the receiver-operating characteristics area under the curve (AUC).

The significant features identified by the best-performing regression model, with four additional clinical variables of interest (HDP, term birth, visit 3 EFW < 3rd percentile, and visit 3 EFW 3–9th percentile), were selected as features for the PGM model. The PGM structure was learned with the R package bnlearn^32^, which provides a Bayesian Information Criterion-based structure search algorithm for the creation of Bayesian networks.^33–35^ The search algorithm explores the entire applicable space of conditional dependencies to discover the optimal network structure for the data. Parameter learning for this optimal network was accomplished using the Bayesian parameter estimation.^36,37^ The PGM graphical structure was also determined, allowing for visualization and greater exploration of risk factor dependencies and the impact of multiple comorbidities on the outcome of composite morbidity.^32,38^ Variables within the PGM that are mutually exclusive (e.g. categories for gestational age at birth) were blacklisted so that, for example, the absence of term birth could not be used to predict preterm birth. Our group has previously published a more technical and in-depth explanation of methods for PGM derivation.^39,40^ Interactions were identified whenever a variable’s direction or magnitude varied according to the presence of another variable to a degree that the 95% confidence intervals did not overlap.

We used stratified 5-fold cross-validation (CV) within the 80% derivation cohort to assess performance for both LR and PGM analysis. The dataset was divided into five folds while maintaining the distribution of classes. Then, four folds of the data were used for training and the last fold was used for evaluation. This process was repeated five times. The metric used for evaluating the models’ performance was the AUC. Final validation consisted of applying the PGM to the validation cohort and assessing its performance and optimal prediction threshold using ROC methodology. The exact PGM was used to estimate the absolute risk (AR) and relative risk (RR) of composite perinatal morbidity across a range of scenarios. The distribution of model risk estimation was obtained using randomly drawn samples of the data with replacement to create 1000 bootstraps. Model estimates were aggregated for all bootstraps to determine their distribution and estimate 95% confidence intervals (CI’s).

Our institutional review board designated this analysis as exempt from oversight based on the definition of human subjects research. Participants gave written informed consent for the IRB approved parent study.

## Results

Of 10,038 potentially eligible participants, we excluded 394 for missing neonatal outcome data and 86 for delivery prior to 20 weeks’ gestation, leaving 9,558 participants who were included in the analysis. Composite perinatal morbidity occurred in 8.2% (n = 783). Table 1 summarizes the demographic and clinical characteristics of analyzed nuMoM2b participants.

### Model validation and applications

**Figure 3:**
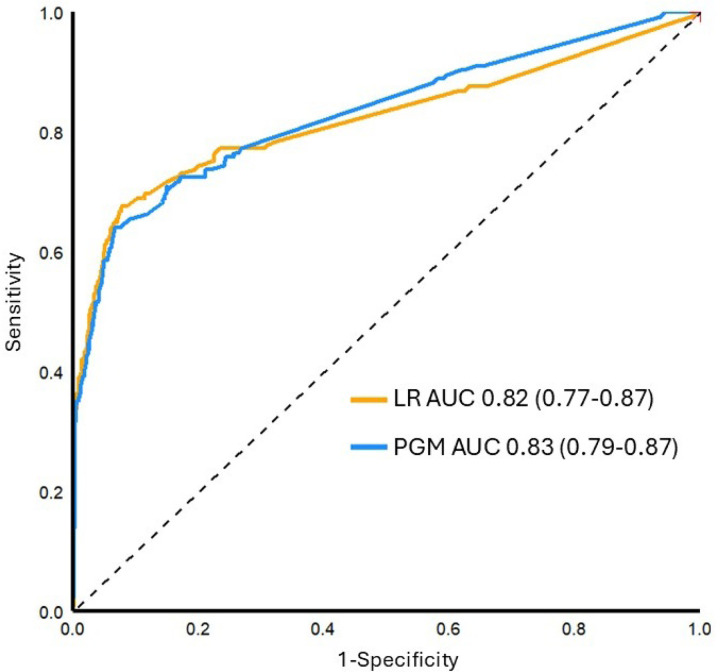
Receiver operating characteristics curves for the PGM and logistic regression model and for prediction of composite perinatal morbidity in the validation cohort. ROC, receiver operating characteristics; PGM, probabilistic graphical model; LR, logistic regression; AUC, area under the curve. AUC 95% confidence intervals shown in parentheses.

When applied to the validation cohort (n = 1,912), the PGM had good performance to identify those at risk for composite perinatal morbidity (AUC 0.83, 95% CI 0.79–0.87), which was similar to the logistic regression model (AUC 0.82, 0.68–0.92, p = 0.8, Fig. 3). Both were similar to the performance estimates from 5-fold cross-validation within the derivation cohort (PGM AUC 0.83, 0.82–0.84; LR AUC 0.82, 0.81–0.83).

**Figure 4:**
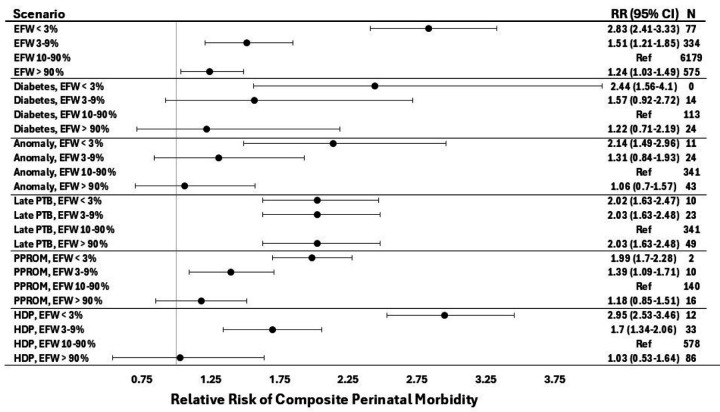
Relative risk of perinatal morbidity conferred by EFW percentile category across a range of obstetric scenarios The RR for each high or low EFW percentile category was compared against EFW 10^th^-90^th^ percentile in the setting of the same clinical scenario, labeled as “Ref.” In the clinical scenarios, “%” denotes percentile. The vertical gray line reflects RR of 1. N’s report the number of participants in the derivation cohort with the associated clinical scenario. Diabetes refers to pre-gestational diabetes. Point estimates are based on the PGM’s maximum likelihood estimates rather than the mean of bootstrapped values, which is why they are not in the center of the confidence intervals. CI, confidence interval; EFW, estimated fetal weight; HDP, hypertensive disorder of pregnancy (any of: gestational hypertension, preeclampsia, superimposed preeclampsia, eclampsia); PTB, preterm birth; PPROM, preterm premature rupture of membranes; RR, relative risk.

However, PGMs provide an advantage over LR approaches by evaluating the complete joint probability distribution of the graphical model, which includes all interdependencies among all variables included in the model. Leveraging this functionality, the PGM identified FGR scenarios in which the risk relationships varied from their typical pattern. In most clinical scenarios we analyzed, the EFW percentile category was associated with a common pattern of risk distribution, in which EFW < 3rd percentile conferred the highest risk, EFW 3–9th conferred an elevated but lesser risk, and EFW > 90th had a risk that was similar to or slightly higher than the reference group of EFW 10–90th percentile (Fig. 4). However, in the setting of late PTB, EFW percentile had a distinct risk pattern from other scenarios, with all EFW percentile categories conferring a similar increase in risk (RR ≈ 2.0, Fig. 4) compared to EFW 10–90th percentile.

**Figure 5:**
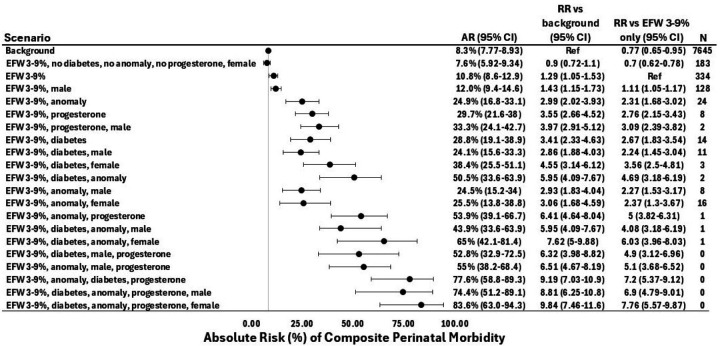
Sequential introduction of clinical factors to non-severe FGR to identify variable combinations that drive composite perinatal morbidity risk. RR columns are based on a given scenario’s comparison to the cohort’s background or to the risk of EFW 3–9^th^ percentile alone (in red). The vertical gray line reflects the cohort’s background risk of composite perinatal morbidity (8.3%). In the clinical scenarios on the left, “%” denotes percentile. Point estimates are based on the PGM’s maximum likelihood estimates rather than the mean of bootstrapped values, which is why they are not in the center of the confidence intervals. Diabetes refers to pre-gestational diabetes. N values represent the number of participants in the derivation cohort who meet the query criteria. AR, absolute risk (expressed as a percent); CI, confidence interval; RR, relative risk; EFW, estimated fetal weight; PTB, preterm birth; PPROM, preterm premature rupture of membranes.

We then used the PGM to estimate risk in several hypothetical scenarios involving EFW 3–9th percentile in combination with multiple other variables, which were sequentially added to identify specific combinations that drive risk. We used the PGM to compute the RR conferred by each scenario in comparison to the cohort background risk (8.3%), and to EFW 3–9th percentile alone. These results are shown in Fig. 5. Among these, the lowest-risk scenario (EFW 3–9th percentile, no diabetes, no progesterone use, female sex) had a perinatal morbidity risk estimate that was no different than the cohort background (RR 0.9, 0.7–1.1) but was lower than EFW 3–9th percentile alone (RR 0.7, 0.6–0.8). The highest-risk of the hypothetical scenarios (EFW 3–9th percentile, preexisting diabetes, fetal anomaly, progesterone use, female sex) had a risk estimate that was nearly 10-fold higher than the cohort background risk (RR 9.8, 7.5–11.6) and 8-fold higher than EFW 3–9th percentile alone (RR 7.8, 5.6–9.9, Fig. 5). The highest risk scenario estimate was based on PGM modeling alone as there were no participants in the derivation cohort meeting these criteria, though there was one such participant in the validation cohort.

### Fetal sex, diabetes, and perinatal morbidity

**Figure 6:**
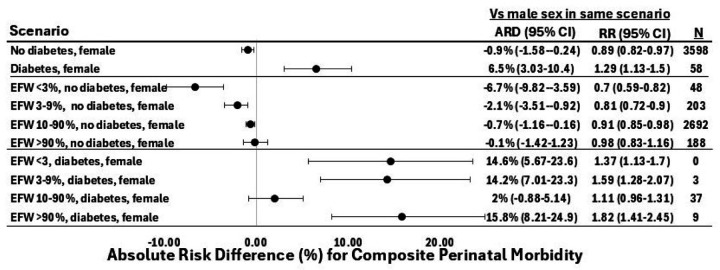
Female sex confers lower perinatal morbidity risk except in the setting of pre-gestational diabetes. In the absence of pregestational diabetes, female sex is protective. In the presence of pregestational diabetes, female sex adds risk. ARD reflects the absolute risk difference (expressed as a percent) for perinatal composite morbidity between female and male sex in the given EFW percentile and diabetes scenarios. The vertical gray line reflects the risk associated with male sex in the same clinical scenario. Point estimates are based on the PGM’s maximum likelihood estimates rather than the mean of bootstrapped values, which is why they are not in the center of the confidence intervals. N values represent the number of participants in the derivation cohort who meet the query criteria. Diabetes refers to pre-gestational diabetes. ARD, absolute risk difference; CI, confidence interval; RR, relative risk; EFW, estimated fetal weight.

This series of PGM queries identified unexpected interactions between fetal sex, preexisting diabetes, and EFW percentile category. When assessed in isolation as well as in most other scenarios, female sex was either protective or had no association with composite morbidity (Supplementary Fig. 2). However, in the setting of preexisting diabetes, female sex was associated with greater risk of perinatal morbidity than male sex (RR 1.3, 1.1–1.5, Fig. 6). When applied to FGR, the associations also varied according to EFW percentile category: in the setting of EFW 3–9th percentile without preexisting diabetes, female sex was associated with lower perinatal morbidity risk compared to male sex (RR 0.8, 0.7–0.9). However, in the setting of EFW 3–9th percentile with preexisting diabetes, female sex conferred elevated risk compared to male sex (RR 1.6, 1.3–2.1). This diabetes-specific association of female sex with morbidity varied by EFW percentile category, constituting a three-way interaction. Female sex in the setting of preexisting diabetes had a similar association with morbidity in the setting of EFW < 3rd percentile (RR 1.4, 1.1–1.7), 3–9th percentile (RR 1.6, 1.3–2.1), and EFW > 90th percentile (RR 1.8, 1.4–2.5), but not in EFW 10–90th percentile (RR 1.11, 1.0–2.5). The RRs and absolute risk differences associated with female sex compared to male sex in FGR and preexisting diabetes are shown in Fig. 6.

### PGM probability estimation for alternate variables

**Figure 7:**
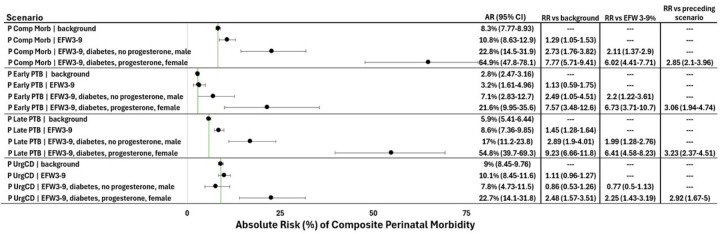
PGM risk estimates for multiple adverse outcomes in FGR in the setting of pre-gestational diabetes vary widely according to progesterone use and fetal sex. The scenarios are framed as probability expressions where “*P Comp Morb | EFW 3–9*” would be written as “the probability of composite morbidity given the presence of EFW 3–9^th^ percentile.” The green lines represent the cohort’s background absolute risk of the associated outcome (composite perinatal morbidity, early PTB, late PTB, or urgent cesarean), allowing for visual interpretation based on confidence intervals that overlap with the background risk estimate. Absolute risks are expressed as percentages. The number of derivation cohort participants meeting criteria for each scenario in order of presentation are: 7645, 334, 11, and 0, respectively. “RR vs background” expresses the relative risk of a given factor or scenario over the cohort’s background risk of the same outcome (green line). “RR vs EFW 3–9%” expresses the relative risk of each scenario over the risk conferred by EFW 3–9^th^ percentile alone. “RR vs preceding scenario” expresses the relative risk of the final scenario over the preceding scenario, in which the only differing factors are progesterone use and fetal sex. All risks (AR, RR) are followed by 95% confidence intervals. Diabetes refers to pre-gestational diabetes. AR, absolute risk; CI, confidence interval; RR, relative risk; EFW, estimated fetal weight; P, probability; PTB, preterm birth; UrgCD, urgent cesarean delivery.

Following our identification of the interaction between fetal sex, preexisting diabetes, and composite perinatal morbidity, we leveraged the ability of PGMs to estimate the probability of any variable in the network without having to derive a new PGM. In other words, PGMs can treat any variable within the model as either a risk factor or the target outcome for probability estimation. We used this PGM function to estimate the probability of three variables initially treated as risk factors for composite perinatal morbidity in various clinical scenarios involving FGR: early preterm birth, late preterm birth, and urgent cesarean (Fig. 7). In the setting of EFW 3–9th percentile and preexisting diabetes, the combination of progesterone use and female sex was associated with significantly increased risk for these four outcomes, compared to no progesterone use and male sex (adverse outcome RR range 2.9–3.23, Fig. 7). When compared to the cohort background, EFW 3–9th percentile alone conferred only a modest risk for these four outcomes (RR range 1.1–1.5), which was somewhat higher in the setting of preexisting diabetes and male sex without progesterone use (RR range 0.9–2.9). However, EFW 3–9th percentile in the setting of a female fetus, preexisting diabetes, and progesterone use conferred much higher risks of adverse outcomes over the cohort background (RR range 2.5–9.2). The combination of progesterone with these variables does not constitute an interaction (Supp. Figure 3) but does highlight the ability of the PGM to identify similar-appearing scenarios that are separated by large differences in risk.

### N of 1 scenarios

Because several of the scenarios outlined in Figs. 4–7 included PGM queries that were represented by few or no participants in the derivation cohort, we assessed the PGM’s performance in rare scenarios occurring fewer than 10 times each in the validation cohort. In the entire nuMoM2b cohort, 3.0% (n = 290) of participants experienced a “unique scenario” consisting of a set of variables (excluding the composite morbidity outcome) not found in any other participant. In the validation cohort, there were 102 such participants (5.3%). Among these N of 1 participants, the PGM performance remained good (AUG 0.81, 0.72–0.90), which remained the case when it was assessed among scenarios occurring fewer than or equal to 5 times (AUC 0.86, 0.81–0.91) or 10 times (0.84 (0.79–0.88).

## Discussion

We developed a PGM with strong performance for composite perinatal morbidity risk estimation and the ability to estimate context-specific morbidity risk in FGR. Starting with a comprehensive assessment of clinical, psychosocial, and experimental ultrasound variables, we found that the set of variables yielding the best overall prediction largely consisted of variables that are routinely ascertained in clinical practice. When using the PGM to explore the risk landscape in FGR based on ultrasound at 22–29 weeks, we found that the PGM captured and quantified an unexpected interaction between preexisting diabetes, fetal sex, and composite perinatal morbidity. The PGM was able to identify scenarios that appear similar clinically but have large differences in morbidity risk. Finally, it maintained good performance among the 102 “N of 1” scenarios in the validation cohort.

The variables retained in the PGM for prediction of perinatal morbidity and mortality are largely consistent with existing literature. The association between progesterone use and increased risk of perinatal morbidity was not surprising since we interpret progesterone use as reflecting increased clinical concern for PTB or miscarriage. While we expected that psychosocial factors would be more informative for risk estimation, the variables retained in the PGM are those most closely associated with composite perinatal morbidity and may be on the causal pathway between social determinants of health and adverse outcomes. If a hypothetical PGM were to include many more variables, psychosocial factors may be retained. However, because PGMs have a practical limit of < 20 variables owing to computational constraints, these variables were not included. Finally, our finding of a diabetes-specific relationship between fetal sex and perinatal morbidity was striking and unexpected. In the PGM, this interaction appeared to vary by EFW percentile category, but the number of participants meeting those query criteria was low or zero, such that those estimates may be less trustworthy. There is an emerging body of literature describing sex-specific responses to maternal metabolic perturbations, including hyperglycemia and obesity, placental gene expression,^41^ fatty acid metabolism,^42^ protein metabolism,^43^ and fetal growth.^44,45^ Findings on the association of fetal sex with perinatal morbidity in gestational diabetes are mixed,^44–46^ and it is uncertain whether the interaction we describe would be expected to generalize to the context of gestational diabetes, which is the focus of most available studies.

To date, investigations of explainable AI in reproductive science have been limited. Broader machine learning (ML) approaches have been used for stillbirth and preeclampsia prediction as well as basic science applications such as multi-omic placental phenotyping, among others.^47–49^ There have been other efforts to utilize explainable ML approaches for prediction of gestational diabetes,^50^ preterm birth,^50^ and composite perinatal morbidity,^51^ though these efforts consisted of developing predictive ML models using traditional approaches, followed by post-hoc application of an algorithm designed to identify the most contributory variables and thereby improve explainability. PGM outputs handle this explainability inherently and without the need for post-hoc interrogation with specialized tools. The explainability of PGMs is apparent in that a given variable’s precise and context-specific statistical contribution to risk is transparently quantified and reported when risk is estimated both with and without the variable of interest. The use of PGMs in reproductive medicine thus far has been limited to optimizing assisted reproductive technologies^52^ and prediction of neonatal pneumonia in the setting of maternal diabetes.^53^

A key advantage of PGMs is their ability to produce relatively precise probability estimates for low frequency or “N of 1” scenarios. Because PGMs can capture and quantify conditional dependencies between interrelated variables, the estimated influence of a given variable is adjusted based on the presence or absence of other factors linked to the given variable via such conditional dependencies. This allows for reproducible and relatively precise probability estimations in the types of “N of 1” scenarios that individually are uncommon but collectively are common in clinical practice. Currently, risk stratification in such scenarios depends on human expert assessment, which is highly flexible but opaque, prone to bias, and influenced by circumstances such as sleep deprivation, mood, and other factors.^9–11^ In contrast, PGMs overcome the opaque and state-dependent pitfalls of human risk estimation thanks to their transparency and reproducibility. PGMs can therefore be used to transparently generate probability estimates, including for rarely occurring scenarios, while human experts can provide both intellectual oversight and patient-centered recommendations.

The U.S. clinical guidelines for management of FGR include the frequency of monitoring and timing of delivery and are based on metrics of FGR severity alone. These guidelines do not take other clinical data into account, allowing clinicians to customize their recommendation using clinical judgement.^54,55^ We identified several clinical scenarios, all involving non-severe FGR (EFW 3–9th percentile) that were separated by up to a 10-fold difference in perinatal morbidity risk (Fig. 5) for which the U.S. guidelines recommend identical management. This reality highlights current gaps and opportunities for personalized risk estimation to reduce prognostic uncertainty of FGR and tailor surveillance and delivery timing plans accordingly.

Our study’s strengths included the comprehensive assessment of variables across domains, including clinical, SDoH, mental health, ultrasound, and quantitative urine toxicology in a large and deeply phenotyped pregnancy cohort. We used a novel explainable AI approach to produce a flexible model with good prediction of composite perinatal morbidity and the potential for integration of genomic and other variables. The cohort is geographically diverse in the U.S. and utilized standardized outcomes ascertainment by centrally trained and certified research personnel.

Limitations include the timing of research ultrasounds at 22–29 weeks and the lack of umbilical artery Doppler assessments, which limit the clinical generalizability, as well as the fact that nuMoM2b, while diverse, is not fully representative of the U.S. population. Progesterone use is an intervention based almost solely on clinical concern and thus likely includes bias, making its associations with morbidity difficult to interpret. We included it, however, as our goal was to derive a PGM using the most empirically informative set of factors for morbidity risk estimation. Finally, our inclusion of variables known only at or after delivery means this model is not useful prenatally. We chose this approach because our objective was not to develop a tool for prenatal use to alter clinical management, but to determine the utility of PGMs to capture and quantify complex risk relationships and identify context-specific risks in FGR. Eventually, this line of inquiry may lead to the development of a tool for prenatal use.

## Conclusions

We successfully developed an explainable AI model with good performance for perinatal morbidity risk estimation and the ability to provide context-specific risk estimates across a range of FGR scenarios, including those that occur at a low frequency. While not yet ready for clinical application, this represents an important proof of concept and demonstration of the potential for PGMs to refine risk estimation in obstetrics.

## Supplementary Material

Supplement 1

## Figures and Tables

**Figure 1:**
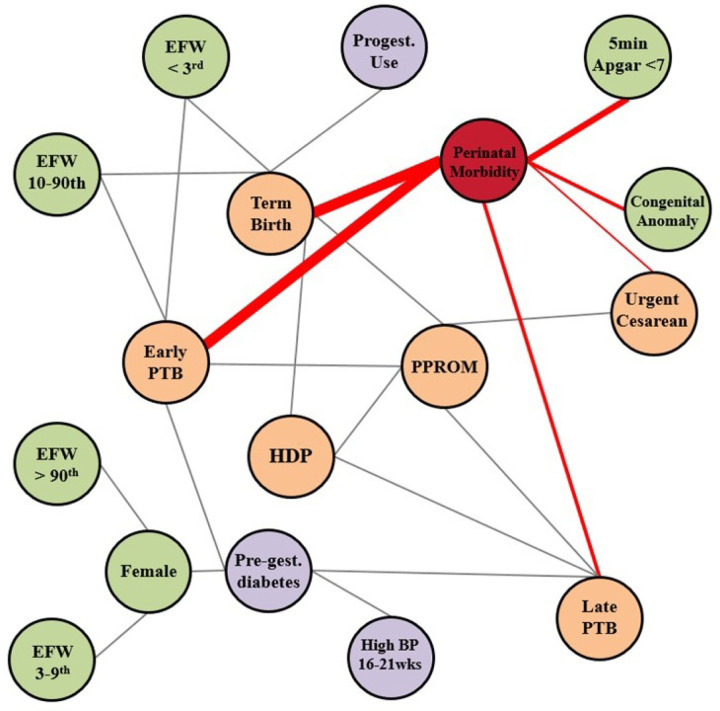
Probabilistic graphical model structure. Nodes in the model represent variables, with lines representing conditional dependencies between variables. The nodes that are directly connected to the perinatal morbidity outcome are connected by red lines, and the thickness of the red lines reflects the strength of association with perinatal morbidity. BP, blood pressure; DM, preexisting diabetes mellitus; EFW, estimated fetal weight; HDP, hypertensive disorder of pregnancy (any of: gestational hypertension, preeclampsia, superimposed preeclampsia, eclampsia); PTB, preterm birth; PPROM, preterm premature rupture of membranes.

**Figure 2:**
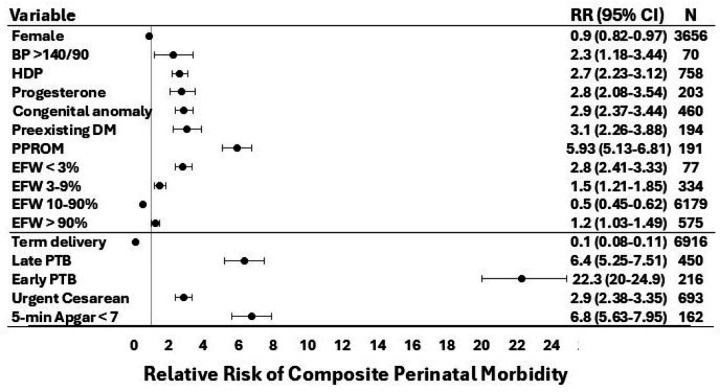
PGM-estimated composite perinatal morbidity risk conferred by individual PGM variables. The vertical gray line reflects the reference (RR=1), based on the cohort’s background risk of composite perinatal morbidity. BP >140/90 was ascertained at visit 2 (16–21 weeks). Factors known prior to delivery (above the horizontal line) are separated from factors only known at or after delivery (below the line). In the EFW categories, “%” denotes percentile. BP, blood pressure; CI, confidence interval; DM, diabetes mellitus; EFW, estimated fetal weight; HDP, hypertensive disorder of pregnancy (any of: gestational hypertension, preeclampsia, superimposed preeclampsia, eclampsia); PGM, probabilistic graphical model; PTB, preterm birth; PPROM, preterm premature rupture of membranes; RR, relative risk.

**Table 1:** Cohort characteristics

Variable	Mean ± SD or %(n)
Maternal age (years)	27.0 ± 5.6
Race	
White	60.3 (5763)
Hispanic	16.8 (1604)
Black	13.8 (1321)
Asian	4.0 (385)
Other	5.0 (481)
Household income*	
< 100% of Fed poverty rate	12.8% (1225)
100–200% Fed poverty rate	11.7% (1114)
> 200% Fed poverty rate	56.8% (5426)
BMI	26.3 ± 6.3
Preexisting diabetes	1.6% (151)
Hypertensive disorders	
Chronic HTN	2.5% (242)
Gestational hypertension	14.2% (1355)
PE without severe features	4.0% (385)
PE with severe features	4.1% (393)
Superimposed PE	0.5% (51)
Eclampsia	0.05% (5)
GA at delivery	
Term	90.5% (8653)
Late preterm	5.8% (556)
Early preterm	2.8% (265)
Female sex	50.9% (4868)
Congenital malformation	5.9% (561)
Composite perinatal morbidity	8.2% (783)
Stillbirth	0.6% (54)
Neonatal death	0.3% (24)
NICU stay > 7 days	4.6% (444)
RDS	3.2% (303)
Mechanical ventilation	2.7% (255)
Confirmed sepsis	0.4% (36)
ROP	0.4% (36)
Seizures	0.2% (19)
NEC	0.1% (12)
Grade 3–4 IVH	0.1% (9)

* Out of n = 7765 with household income available.

Based on feature selection from the 80% derivation cohort (n = 7,645), the final list of variables in the PGM included the following (Table 2): maternal variables: progesterone use, pre-existing diabetes (type 1 or 2), and BP > 140/90 at visit 2 (16–21 weeks); obstetric variables: gestational age at birth (term, late preterm, early preterm), HDP (as defined above), preterm premature rupture of membranes (PPROM), urgent cesarean; and fetal/neonatal variables: sex, presence of any congenital anomaly, 5-minute Apgar < 7, and EFW percentile (< 3rd, 3–9th, 10–90th, > 90th ) at visit 3 (22–29 weeks).

**Table 2:** Summary of variables included in the PGM

Variable	%(n)
Preexisting diabetes	1.6% (1251)
Progesterone use	2.6% (246)
Female sex	50.9% (4868)
Congenital malformation	5.9% (561)
Visit 2 BP > 140/90	0.8% (81)
Visit 3 EFW percentile	
<3rd	1.0% (97)
3–9th	4.3% (408)
10–90th	81.0% (7745)
>90th	7.3% (696)
PPROM	2.6% (246)
Hypertensive disorders of pregnancy	10.0% (952)
GA at delivery	
Term	90.5% (8653)
Late preterm	5.8% (556)
Early preterm	2.8% (265)
Urgent cesarean	9.1% (871)
5 minute Apgar < 7	4.0% (382)

The PGM graphical structure is shown in Fig. 1. PGM variables had low missingness, with all variables missing less than 2% of data points except for visit 3 EFW (6.4%) and visit 2 BP > 140/90 (4.9%, Supplemental Table). Variables with direct connections to perinatal morbidity included early preterm birth, late preterm birth, term birth, urgent cesarean delivery, congenital anomaly, and 5-minute Apgar < 7. Of note, only term birth conferred a decreased risk of the outcome, while all other directly connected variables conferred an increased risk of composite perinatal morbidity (Fig. 1). The PGM-estimated risk relationships for each variable with composite perinatal morbidity are shown in Fig. 2 and were similar when assessed in the setting of FGR (Supplementary Fig. 1).

## Data Availability

NuMoM2b study data are available on request at the Data Access Specimen Hub (DASH), hosted by the *Eunice Kennedy Shriver* National Institute of Child Health and Human Development, https://dash.nichd.nih.gov.

## Abbreviations

AI: artificial intelligence.
AR: absolute risk.
ARD: absolute risk difference.
AUC: area under the curve.
BP: blood pressure.
CI: confidence interval.
CV: cross-validation.
DM: (preexisting/pregestational) diabetes mellitus
EFW: estimated fetal weight.
FGR: fetal growth restriction.
GA: gestational age.
HDP: hypertensive disorder of pregnancy.
HTN: hypertension.
IVH: intraventricular hemorrhage.
LR: logistic regression.
ML: machine learning.
NEC: necrotizing enterocolitis.
NICU: neonatal intensive care unit.
PE: preeclampsia
PGM: probabilistic graphical model.
PPROM: preterm premature rupture of membranes.
PTB: preterm birth.
RDS: respiratory distress syndrome.
ROP: retinopathy of prematurity.
ROS: receiver operating characteristics.
RR: relative risk.
UrgCD: urgent cesarean delivery.
