# Supplementary material for: AI-based analysis of fetal growth restriction in a prospective obstetric cohort quantifies compound risks for perinatal morbidity and mortality and identifies previously unrecognized high risk clinical scenarios
Source: Res Sq. 2024 Dec 16:rs.3.rs-5126218. Preprint. [Version 1] doi: 10.21203/rs.3.rs-5126218/v1 (PMC11702817; doi:10.21203/rs.3.rs-5126218/v1)
Supplement: Supplement 1 [file NIHPPRS5126218v1-supplement-1.pdf]

Supplementary Files

This is a list of supplementary files associated with this preprint. Click to download.

- Suppfig1.jpg
- Suppfig2.jpg
- SuppFig3.jpg
- Supptablesmissingness.docx
